# A scenario analysis of the future residential requirements for people with mental health problems in Eindhoven

**DOI:** 10.1186/1472-6947-11-1

**Published:** 2011-01-06

**Authors:** Joyce JPA Bierbooms, Inge MB Bongers, Hans AM van Oers

**Affiliations:** 1 Tilburg University, Department Tranzo, P.O. Box 90153, 5000 LE Tilburg, the Netherlands; 2 GGZ Eindhoven en de Kempen, P.O. Box 909, 5600 AX Eindhoven, the Netherlands; 3 IVA, Tilburg University, P.O. Box 90153, 5000 LE Tilburg, the Netherlands; 4 National Institute for Public Health and the Environment (RIVM), P.O. Box 1, 3720 BA Bilthoven, the Netherlands

## Abstract

**Background:**

Despite large-scale investments in mental health care in the community since the 1990 s, a trend towards reinstitutionalization has been visible since 2002. Since many mental health care providers regard this as an undesirable trend, the question arises: *In the coming 5 years, what types of residence should be organized for people with mental health problems? *The purpose of this article is to provide mental health care providers, public housing corporations, and local government with guidelines for planning organizational strategy concerning types of residence for people with mental health problems.

**Methods:**

A scenario analysis was performed in four steps: 1) an exploration of the external environment; 2) the identification of key uncertainties; 3) the development of scenarios; 4) the translation of scenarios into guidelines for planning organizational strategy. To explore the external environment a document study was performed, and 15 semi-structured interviews were conducted. During a workshop, a panel of experts identified two key uncertainties in the external environment, and formulated four scenarios.

**Results:**

The study resulted in four scenarios: 1) Integrated and independent living in the community with professional care; 2) Responsible healthcare supported by society; 3) Differentiated provision within the walls of the institution; 4) Residence in large-scale institutions but unmet need for care. From the range of aspects within the different scenarios, the panel was able to work out concrete guidelines for planning organizational strategy.

**Conclusions:**

In the context of residence for people with mental health problems, the focus should be on investment in community care and their re-integration into society. A joint effort is needed to achieve this goal. This study shows that scenario analysis leads to useful guidelines for planning organizational strategy in mental health care.

## Background

In the field of specialized mental health care in the Netherlands there are two main types of care for people with (complex) mental health problems. There is inpatient care, where patients stay in a psychiatric hospital unit (institutional care), or in a small-scale residential unit in the community with supervision and support from a mental health care provider. There is also outpatient care, where patients live independently and receive ambulatory treatment or care (see Table 1 for terminology). About 90% of patients in Dutch mental health care receive outpatient care [1]. In the 1990 s the focus of mental health care in the Netherlands shifted from mainly institutional care to the extending of community care (small-scale residential care and outpatient care). Stimulated by the Dutch government, mental health care organizations gradually reduced the number of conventional inpatient beds in return for a growth in small-scale residences and outpatient care (deinstitutionalization).

**Table 1:** Terminology

Inpatient care	Care that is supplied while residence is provided by the mental health care supplier at a central location or in society (see 'small-scale residential care').
Institutional care	Inpatient care at a central location.

Small-scale residential care	Care that is supplied while residence is provided in the community by the mental health care supplier.

Outpatient care	Ambulatory care.

Conventional inpatient beds	Beds in the institution for regular intramural care; institutional care.

Forensic beds	Beds in the institution for specialized forensic care.

Places for supervised and supported housing	Rooms or apartments that are available in forms of residence outside the institution (in the community), with supervision and support.

Community care	Small-scale residential care or ambulatory care aimed at integration of mental health patients into society.

Deinstitutionalization	A development of more outpatient care outside the institution and reduction of inpatient care.

Reinstitutionalization	A development leading to more inpatient (mental) health care after a period of predominantly outpatient care.

Table 2 shows that deinstitutionalization has stagnated since 2002 [1-3]. A recent report by the Dutch Association for Mental Health and Addiction Care shows a 3.6% growth in the number of patients receiving inpatient care between 2005 and 2007 [1]. Similarly, reports by the Netherlands Institute of Mental Health and Addiction show a 5.6% increase in inpatient days in mental health care between 2002 and 2005 [3]. Although supervised and supported residences show an increase between 2002 and 2006, Priebe et al. also report a 6.3% increase in the number of conventional inpatient beds [2].

**Table 2:** Inpatient and outpatient care facilities per 100,000 inhabitants in the Netherlands between 1990 and 2007

*Per 100,000 persons*	*1990*	*1993*	*1998*	*2002*	*2005*	*2006*	*2007*
Netherlands Institute of Mental Health and Addiction, 2007 (based on data from the Dutch Healthcare Authority)*							
Conventional inpatient beds	-	152	141	124	131	-	-
Supervised and supported housing places	-	26	34	38	51	-	-
Outpatient contacts	-	26,780	30,305	31,182	57,551	-	-

Priebe et al., 2008 (based on data in Psychiatric Case Registers)**							
Conventional inpatient beds	161	-	-	128	-	136	-
Forensic beds	5	-	-	11	-	14	-
Places for supervised and supported housing	25	-	-	40	-	51	-

Dutch Association for Mental Health and Addiction Care, 2009*							
Inpatient beds (conventional + supervised and supported housing)	-	-	-	-	165	171	171
Outpatient contacts	-	-	-	-	57,592	63,605	73,337

*calculated based on original numbers

** original numbers in research paper

Priebe et al. cite several possible explanations for this emerging reinstitutionalization: greater morbidity related to urbanization, changing lifestyles and drug use, an increase in risk aversion, a decrease in informal support, a strategy of healthcare providers to invest in (relatively budget-safe) inpatient care, and the tendency of health insurance companies to move healthcare costs for complex groups away from private insurance to the social care sector (provided in the Netherlands under the Social Support Act or Exceptional Medical Expenses Act) [2].

This reinstitutionalization is an undesirable trend in relation to the policy vision that mental health care in the community should be stimulated [4]. Therefore, mental health care providers have been planning policy interventions to restrict this trend. The question they face in relation to a new policy period, in which choices must be made concerning their residential facilities, is: *In the coming 5 years, what types of residence should be organized for people with mental health problems? *This question also arose at *Stichting Geestelijke Gezondheidszorg Eindhoven en de Kempen *(GGzE), a mental health care provider in Eindhoven. With a population of 212,000, Eindhoven is the fifth largest city in the Netherlands. Because of the regional function of GGzE, it has a working area of 525,000 people (Eindhoven and surrounding towns and villages), in which institutional care increased by almost 6% between 2006 and 2008.

Initially, a projection was made to estimate the number of patients that could be expected in 5 to 10 years. However, this demographic projection did not provide a reliable basis. Apart from demographic changes, the mental health care sector is also faced with socio-economic changes and developments in society. These developments can have considerable impact on people with mental health problems, and consequently on the options of mental health care providers regarding the types of residence for their patients. Because this leads to much *uncertainty *about the characteristics of future residences for mental health care, an additional qualitative study was needed to explore the influence of these developments.

A method for exploring the many aspects of future developments, in which uncertainty plays an essential role, is offered by *scenario analysis*. The essence of scenario analysis is to acquire a better understanding of the external environment, to create pictures of possible futures, and to enhance policy planning based on these pictures [5]. In the Netherlands, several organizations in the social sector perform scenario studies, including the Dutch Network for Futures Research, Strategy Development and Health Care Innovation [6], the 'Public Health Status and Forecasts' of the National Institute for Public Health and the Environment [7], the Netherlands Institute for Social Research [8], and, in the mental health care sector, the Netherlands Institute of Mental Health and Addiction [9]. All these organizations perform *macro-level *studies on scenarios in healthcare. In the profit sector, several organizations use scenario analysis on a smaller scale as a tool for planning organizational strategy. Since the successful use of the scenario approach by Shell at the time of the oil crisis, various other organizations (e.g. the car industry) have adopted the method [10-12]. This suggests that scenario analysis may also contribute to better strategy planning at a regional level in the healthcare sector. Therefore, the present study used scenario analysis to gain more insight into the need for different types of residence in mental health care in Eindhoven. This study was carried out by GGzE, in cooperation with public housing corporations and the Eindhoven Local Authority.

The main questions are:

1. *What are realistic scenarios concerning different types of residence in mental health care in Eindhoven?*

2. *What are the implications of these scenarios for GGzE, public housing corporations, and local government?*

3. *What guidelines does the scenario analysis offer for planning organizational strategy?*

## Methods

### General background

Unlike in the past, when strategy planning was based primarily on quantitative extrapolations, qualitative explorations (resulting in scenarios) regarding future developments have nowadays become more common. Although mathematical elaboration can be part of scenario analysis [13], explorations based solely on quantitative analysis often fail, and can lead to wrong assumptions about a future situation [10,14]. In scenario analysis the most important uncertainties are charted as a starting point for developing scenarios. This method can help an organization to develop policy plans and anticipate future developments [5,15-20]. This does not mean that the future is defined, but that several possible futures are mapped, each of which is realistic but not certain [15-17,21].

### Scenario analysis approach

Over the years, there have been several authors who describe scenario analysis as a method for strategic planning, e.g. Van der Heijden [15,17], Postma & Liebl [16], and Wright et al. [5]. Probably one of the best known is the scenario analysis method of Van der Heijden [15]. In his book '*Scenarios: the art of strategic conversation' *the author describes both an inductive and a deductive approach to scenario development. The deductive method is described as working from the general to the more specific, whereas the inductive method uses a bottom-up approach. The deductive method, more than the inductive method, aims at thinking beyond what can be reasoned, and is more likely to identify uncertainties [15]. The deductive method of scenario analysis embodies, in summary, the following steps: 1) an exploration of the external environment; 2) the identification of key uncertainties; 3) the development of scenarios; 4) the translation of scenarios into guidelines for planning organizational strategy [15]. Different aspects of these stages of scenario analysis can also be found in other literature, e.g. Ringland [11], van Asselt [12], Postma & Liebl [16]. Dammers [18] and Schoemaker [22].

**Figure 1:**
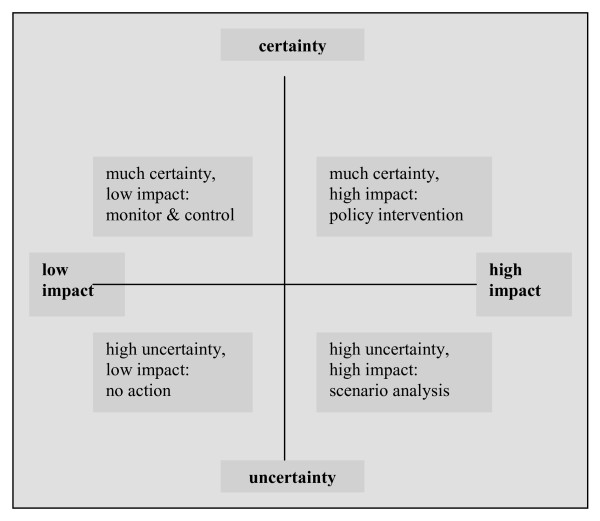
**Step 2: the identification of key uncertainties**.

**Figure 2:**
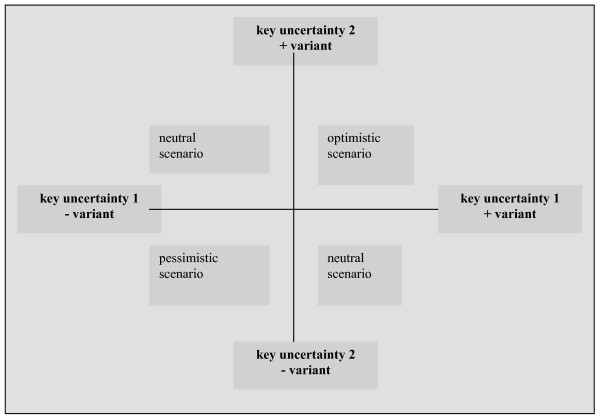
**Step 3: development of scenarios**.

The first step is to explore the external environment and identify which certain and uncertain developments confront the organization. This exploration can include a document study of relevant reports and websites, an interview with stakeholders, or both. Exploring the external environment should result in a set of uncertainties concerning future policy of the organization [15,17,18]. The second step is to narrow down the external environment to two key uncertainties, based on the level of uncertainty and the impact on organizational policy issues [11,12,15-18]. A method for choosing key uncertainties is to display the uncertainty items on two axes, one determining the level of (un)certainty and the other representing the level of impact [11,15]. Figure 1 shows that the items which are seen as highest on the uncertainty and impact scales represent the key uncertainties that form the basis for scenario analysis. Items that are seen as high on uncertainty and low on impact are of minor importance to the organization's success, and are therefore in the 'no action' quadrant. Furthermore, items that are considered low on uncertainty and high on impact are important issues that are transparent to the organization and can therefore be anticipated in policy interventions. Finally, low uncertainty-low impact items are also transparent, but of minor importance and should therefore be monitored and kept under control (Figure 1) [23]. The scope of our study is limited to scenario analysis. In the third step, the two key uncertainties are used as the axes in a new diagram, giving rise to four scenarios [15,18,22] (Figure 2). In the fourth step, the four possible futures are discussed by a group of stakeholders. For each of the scenarios, the effect of the scenario on a given subject is described, as well as what will happen if no action is taken. Based on this discussion, policymakers are offered guidelines for the further consideration of their organizational strategy planning [5,15,16]. Depending on the scope of the study, these strategies are tested against each of the scenarios that has been developed [15].

### Approach in Eindhoven

The aim of this scenario analysis was to provide guidelines for strategic planning for GGzE and its cooperating partners concerning residence for people with mental health problems in Eindhoven. To perform the study, we used the steps of the deductive scenario analysis method described by Van der Heijden [15].

#### Exploring the external environment (step 1)

In the scenario analysis in Eindhoven, the external environment was mapped by a document analysis, followed by 15 interviews with a total of 20 participants. Both the document analysis and the interviews were guided by a framework of themes. These themes were selected on the basis of the notion that future scenarios are a collection of events that all have a certain degree of probability of occurring [10,14]. In their Public Health Forecast, the National Institute for Public Health and the Environment refers to demographics, economics, social and cultural developments, technology, and space as the driving forces of external developments [7]. To perform step 1 of the scenario analysis we chose to use these driving forces as a guideline for determining the covering themes of our framework. Furthermore we included policy related developments as a theme and combined cultural developments, technology, and space into one theme (social developments). For exploring the external environment, this leads to the following framework of developments related to:

• demographics

• socio-economics

• society

• policy

### Document analysis

In the Netherlands, the national associations that engage in uncovering developments in society and future scenarios are the *Netherlands Institute for Social Research, Statistics Netherlands*, the *Netherlands Bureau for Economic Policy Analysis*, and the *National Institute for Public Health and the Environment*. These organizations are appointed by law to advise the Dutch Ministry of Health, Welfare, and Sport on national healthcare and welfare issues. We therefore chose to analyze reports from these associations in order to arrange our picture of the most recent developments concerning society as a whole. We then gathered more specific information on mental health care from the *Dutch Association of Health and Addiction Care *(the umbrella organization for the sector), and the *Netherlands Institute of Mental Health and Addiction*, (the expertise centre for mental health and addiction care in the Netherlands). To complete the document analysis, we also looked at reports by Aedes-Actiz, a branch organization concerned with residence and care for special groups in society.

We coded the information on the basis of the themes we selected before starting our exploration (step 1). The information found in the document analysis, using the themes as search criteria, led to a further specification of our framework.

### Interviews

The interviews were held with representatives from mental health care providers (7), public housing corporations (5), local government (2), financiers (2), patients' representatives (2), and patients' family organizations (2). A semi-structured interview was conducted, guided by our framework. The interviews were recorded and transcribed. The information was coded on the basis of the framework, and worked out in more detail using the data that was obtained from the interviews. In a workshop, a panel of experts further discussed the interview results. The panel, consisting of experts who were also involved in the study as interviewees, were given the interview data and asked to agree which themes were significant for further scenario building.

#### Identification of key uncertainties and the development of scenarios (step 2 and 3)

In the interviews we focused on people's views of the present situation and developments they expected in relation to the position of people with mental health problems in society and the consequences for the provision of residences. The result of step 1 is a list of uncertainties deduced from the document study and interviews. These uncertainties were used in a workshop with a panel of 11 experts. These were in policy or management functions with: mental health care providers (6), public housing corporations (3), and local government (2) in Eindhoven. We specifically approached these organizations to participate in the workshop because of their shared responsibility for residence provision and policy for mentally vulnerable people in Eindhoven. The dominance of the mental health care providers' discipline within the group was controlled by using a facilitator who ensured that every member of the panel was given the opportunity to participate in the discussion, and by dividing people from different disciplines equally within the subgroups that worked on the scenarios during the session.

By means of a two-axis system (Figure 1), two key uncertainties affecting residence for people with mental health problems in Eindhoven were identified (in 3 subgroups) from the list of uncertainties (step 2). Figure 2 was then used by the subgroups to compile four scenarios. The scenarios represent a first draft of the expectations that stakeholders have of the future and the implications that these scenarios have for a specific organizational strategy. In this scenario analysis, policymakers of organizations concerned with residence for people with mental health problems in Eindhoven, jointly outlined four scenarios that provide guidelines for actions to optimize the provision of residences (step 3).

#### Translation of scenarios into guidelines for planning organizational strategy (step 4)

This scenario outline was further defined using the plenary discussion that followed the initial compiling by the subgroups in the workshop. The different aspects of the scenarios were discussed, which led to the recognition that several actions needed to be taken in strategy planning in order to anticipate future developments. The scenario analysis and discussion about the impact and consequences of the scenarios provided the panel with guidelines for strategic planning. The actual drawing up of strategic plans, and the testing and development of these plans for each of the scenarios [15], did not take place within the scope of our study.

## Results

### Exploring the external environment (step 1)

The external environment was explored using the framework as described in the Methods section. It can be characterized by demographic, socio-economic, social, and policy topics.

Demographic topics that were found in the document study were aging, immigration, and family composition [24]. During the coming 30 years, the average age of the population of the Netherlands will be increasing, mainly due to the baby boom of the 1940s/1950s [24]. An increasing number of the elderly, combined with a decreasing labor force, will have consequences for healthcare demand and supply. Various technological developments in healthcare will partly compensate for a smaller working population. These will meet the growing wish of people to be self-reliant despite a physical or mental handicap. Furthermore, the document study resulted in a set of topics concerning socio-economic developments, such as income, work, and education [7], which will have their effect on future care consumers and also on the match between supply and demand in healthcare.

Due to new market forces, the (mental) healthcare sector has major concerns about the financial resources that will be available in the near future for adequate residence, care, and treatment for patients with mental health disorders. This implies that a larger demand may be made on society. At the same time, society is changing rapidly: in the coming years individualism in society will increase further, which will diminish social cohesion [25]. In addition, there will be greater freedom of choice, making individuals more critical and assertive about their needs and wants [25]. Acceptance, integration, and the use of technology in healthcare are issues that are largely dependent on these developments in society.

The interviews showed that, in general, experts believe that this means that people with mental health problems will be easily stigmatized and excluded from society, which places more pressure on intramural facilities. The reinstitutionalization trend is already visible in the Netherlands: from 2002-2006 there was a 6% increase in the number of psychiatric hospital beds [2]. The document study and interviews resulted in a set of uncertainties concerning the external environment (Table 3).

**Table 3:** List of uncertainties

Demographics	Socio-economics
- co-morbidity of mental and somatic problems	- available labor force
- different care demand of the elderly in the future	- freedom of choice for mental health care patients
- increasing care demand in autism	- empowerment of mentally vulnerable people
- independent living for the elderly	- role of relatives in empowerment of mentally vulnerable people
	- accessibility of mental health care
**Society**	**Policy**
- acceptance of mental health care patients in the community	- availability of sufficient financial resources
- effects of technological developments	- differentiation in supply
- independent living for mental health care patients	- future of the Exceptional Medical Expenses Act
- possibilities of successful integration of people with mental health problems	- market forces in (mental) health care
- stigmatization of mental health care	- patient flow possibilities
- social tolerance level	- political developments
- reaching foreign patients	- Social Support Act for people with mental health problems
	- separation of residence and care
	- tuning and cooperation between network partners

### The identification of key uncertainties (step 2)

**Figure 3:**
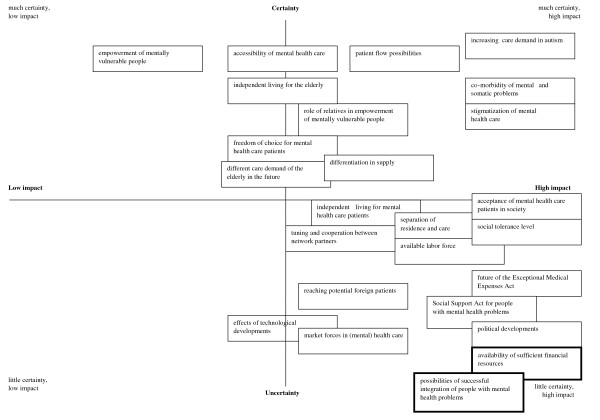
**Plot of uncertainties in the external environment resulting in key uncertainties**.

Using the list of uncertainties (Table 3), two key uncertainties were identified: the *availability of financial resources *for mental health care and the *possibilities for successful integration *into society. Figure 3 shows how the different uncertainties that emerged from exploring the external environment (step 1) were placed on two axes, and resulted in two key uncertainties as the basis for four scenarios.

### The development of scenarios (step 3)

**Figure 4:**
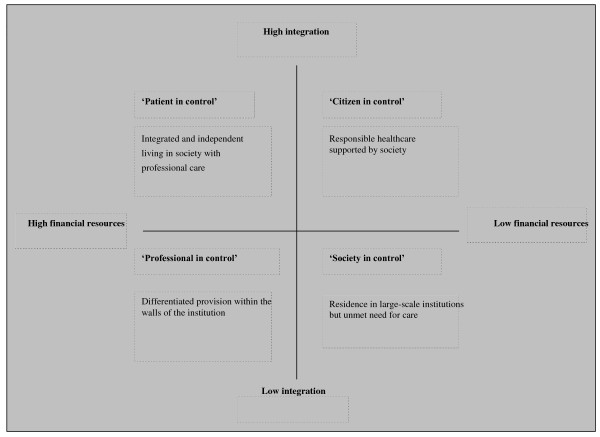
**Scenarios for residence for people with mental health problems in Eindhoven (Eindhoven, 4 November 2008)**.

To find four scenarios that are realistic (but still uncertain), the two key uncertainties were used as scales in a system of axes that was used to develop scenarios. Based on a discussion of the implications of the different possible combinations of key uncertainties on types of residence needed for people with mental health problems, the panel compiled four scenarios (Figure 4):

1. Integrated and independent living in the community with professional care (high on financial resources, high on integration)

2. Responsible healthcare supported by society (low on financial resources, high on integration)

3. Differentiated provision within the walls of the institution (high on financial resources, low on integration)

4. Residence in large-scale institutions but unmet need for care. (low on financial resources, low on integration)

#### Scenario 1: Integrated and independent living in the community with professional care

A high level of integration into society of people with mental health problems is an important policy goal of mental health care providers, public housing corporations, and local government. Successful integration of people with mental health problems enhances the wellbeing of these people, and may lead to a lower need for professional care. Integration also advances people's possibilities of being self-reliant and of living independently or in a group. In this scenario financial resources can provide for highly differentiated and specialized outpatient treatment by a mental health care provider.

#### Scenario 2: Responsible healthcare supported by society

In a scenario where integration is high and financial resources in the mental health care sector are low, the demand for professional mental health care could decrease. This would be due to voluntary care from a patient's direct environment, which 'takes over' certain aspects of care that would otherwise need to be delivered by (costly) professional facilities. Furthermore, mental health problems may be partly prevented, symptoms may be reduced because of the individual's integration into society, and negative influences on wellbeing and functioning may be reduced through integration into society. This scenario would mean that there is limited need for professional care. Obviously, there is also a group of people with mental health problems with complex psychiatric disorders who (also in this scenario) require professional treatment and (residential) care. Nevertheless, should this scenario become a reality, there would be a large increase in demand for independent housing facilities and small living facilities among those requiring mental health care.

#### Scenario 3: Differentiated provision of healthcare within the walls of the institution

In scenario 3, integration has failed, which will further stimulate the pressure on intramural facilities within mental health care institutions. People with mental health problems will be dependent on the 'safe' environment of the institution. Concentrated within the institution, a large range of differentiated customized care is available, with separate facilities for each group. There will probably be a reduced need for independent housing facilities outside the institution, since society does not accept their use. In summary, in this scenario there will be an increase in demand for professional care in an intramural setting, and the mental health care sector will have the financial resources to amply provide it.

#### Scenario 4: Residence in large-scale institutions but unmet need for care

In this feared scenario of both a low integration level and a low availability of financial resources in mental health care, patients' needs and quality of life are no longer central in planning supply. The lack of social integration of people with mental health problems will stimulate the need for residential facilities in a safe and protected environment. In these facilities there will be too few staff to deliver adequate care. Customized care will become a rarity. Independent and normalized living for people who cannot manage on their own will have no place in this scenario. The availability of professional support, care, and treatment will be limited and only in large-scale institutions.

### Translation of scenarios into guidelines for planning organizational strategy (step 4)

In the final step, the panel translated the results into guidelines for organizational strategy. Examining the four scenarios, the panel formulated two key strategies: investing in financial and social security, which means institutional, predominantly inpatient care facilities, or taking some acceptable risks and investing in the integration into society of people with mental health problems.

Market forces have increasingly been driving healthcare providers to focus on financial resources. In the Netherlands, since 2006, the healthcare financing system has changed from budget-based costing towards performance-based costing. The limited budget compels mental health care providers in the Netherlands to negotiate every year with financiers about healthcare procurement. Consequently, a scenario in which financial resources are high and integration into society is successful (scenario 1), initially seemed the most positive scenario to the panel. In this scenario the financial resources are abundant, making it possible to offer broad and differentiated care programs, including specialized mental health care and a wide range of options for residential facilities. However, after careful consideration of the consequences and implications of this scenario, the panel found that several negative outcomes can be expected. When there is an abundance of financial resources this can lead to a substantial supply of professional care, which can have negative consequences at the integration level. Excessive professionalization of healthcare for people with mental health problems living in the community can lead to further stigmatization which, as a result, would again isolate them from society. The panel concluded that that would lead to scenario 3 (*Differentiated provision of healthcare within the walls of the institution*). With current budget cuts in the healthcare sector scenario 1 seems a risk. Also, financiers - under the pressure of a limited budget - tend to invest in the financially more solid intramural facilities [2]. Choosing financial security restricts patients in their opportunity for recovery and equal citizenship. Each citizen has the right to an agreeable living environment, to perspectives, to freedom of choice and participation in society [4]. This demands an extra effort on behalf of people with mental health problems. After this discussion in the workshop, the panel concluded that strategies should be aimed at trying to fulfill scenario 2 (*Responsible healthcare supported by society*) [26].

## Discussion

### From scenarios to organizational decision making

In this case study, the panel unanimously considered 'integration' to be the basis for their future strategy planning. Without the scenarios, policy would probably be primarily aimed at gaining substantial financial resources. Although the latter is important, the scenarios were an eye-opener, and showed the panel that more focus on integration and participation in society of people with mental health problems is probably better realized under the pressure of a limited budget. This does not imply the need for new policy plans for the mental health care branch, because it has been a point of discussion for the last few decades. Paradoxically, care in the community has been the vision of the sector for several years, but it has not yet been successfully taken up in all of the sector's policy. To achieve this, there should be more cooperation between public housing corporations, local government, and collaborating partners. The study also shows the difficulty of achieving integration, especially in view of the current financial pressure. Besides financial uncertainty, there is the question of a changing society. Because of individualism, changing lifestyles, and the falling tolerance level, much effort is needed to achieve a situation in which people take responsibility for each other. Mental health care providers, public housing corporations, and local government in Eindhoven concluded that they have to consider these developments in relation to their policy goals.

Achieving integration is, to a large extent, one of the primary policy goals of care providers, local government, and public housing corporations. Successful integration into society advances the wellbeing of people with mental health problems, and should decrease the need for professional healthcare. This enhances the possibility of achieving independent or group living in a normal community environment as the dominant type of residence. Conversely, reduced financial resources, should act as an incentive for society to take responsibility. Healthcare providers, together with public housing corporations and local government, aim to invest in making people aware of this responsibility. When achieved, this could mean that participation of mentally vulnerable people in society is enhanced with less financial expenditure. However, this demands considerable action, which is represented in the 4-year policy plans of GGzE: investing in "*social support systems, separation of residence and care (e.g. by using home automation facilities), effective spending on daycare, reaching closely involved people, and investing in chain care*". There should be pressure against further extension of residential care. This does not imply that inpatient care has no benefits or should gradually disappear because a large group of people will not be able to live in the community on their own. For this latter group, inpatient care is needed and should be available in an agreeable and safe environment. Nevertheless, the vision in Eindhoven is that the goal should be to treat people as much as possible with ambulatory care, and to institutionalize only if there is no possibility for the patient in the community. This changes the way of thinking about inpatient care from "yes, if..." to "no, unless...".

### The usefulness of scenario analysis as a method for policy planning

#### Benefits

Scenario analysis has proved to be a useful tool for providing guidelines for organizational decision making in healthcare. That has been shown in this case study in Eindhoven. First, it helped to achieve a shared mindset on the central issue of residence for people with mental health problems. Second, it provided a structure in which uncertain developments concerning this topic were easily exposed by considering a broad range of uncertainties that were systematically narrowed down to two key uncertainties. Third, by means of deducing the key uncertainties and then compiling scenarios, the participants in the workshop achieved a better understanding of the alternative future possibilities. Fourth, this scenario analysis resulted in guidelines for future policy plans. These plans have a good potential for success, since their foundation was established by various stakeholders in a shared setting. Finally, this scenario analysis provided a method for identifying the essence of the central theme, which in this case led to four scenarios with guidelines for future policy on residence in mental health care. Drawing on the jointly established view on possible futures for people with mental health problems in Eindhoven, GGzE was able to make strategic plans in the specific area of residence in mental health care. The managing director of GGzE Psychiatry (adults) and Geriatrics division has used this scenario analysis in the division's policy plan for the next 4 years. The key message in this policy plan is recovery and equal citizenship.

#### Generalizability

This study in Eindhoven has shown that it is possible to translate macro-level developments to organizational policy planning using systematic scenario analysis. While current scenario studies are mainly performed at a national level, the method has proved useful at a regional level. Uncertainties can be made transparent at different levels and for specific themes using a uniform method. It is essential to include all necessary stakeholders in the scenario analysis, and to translate macro-level developments to the appropriate level of discussion.

### Important remarks about the use of scenario analysis

#### Scope of scenarios

By focusing on two key uncertainties, the range of topics that have to be taken into account is reduced considerably, and this may involve the risk of ignoring important policy topics. It is therefore essential to involve both certain and uncertain developments in the scenarios. Furthermore, identifying the uncertainties does not mean we have a complete view of all possible futures. Besides the uncertainties, there are the 'unknowables' that are naturally missing from our scenarios (van der Heijden, 1996 en Postma & Liebl, 2005). This means that there will always be a certain number of unexpected and unanticipated events affecting an organization's business strategies, even when based on scenario analysis. Also, rapid changes in society may imply that the scenario analysis is easily outdated. Therefore, scenario analysis should not be a single operation, but an iterative process which has a fixed place in the policy cycle of a healthcare provider and its associates.

#### Testing strategy plans

Van der Heijden [15] explicitly discusses the need to test strategy plans against the many different aspects brought forward by the scenarios. In our study, we performed the steps from exploring the environment to interpreting the different scenarios. However, to conduct scenario analysis according to the method Van der Heijden [15] proposes, a final step should be taken, involving a simulation of the scenarios in which future business strategies are tested and further developed. This would enable the full exploitation of the benefits of scenario analysis.

#### Assessing all scenarios

After developing the scenarios, a discussion took place to work out tangible guidelines for the planning of future strategy for the residence and care of people with mental health problems in Eindhoven. This resulted in the panel focusing on scenario 2 (*Responsible healthcare supported by society*) as the best basis. However, in scenario analysis it is important to assess all aspects in the different scenarios before developing strategy plans. It is therefore essential to scenario analysis to prevent focusing on a single scenario during the process.

### Methodological limitations

A practical limitation of this scenario analysis method is that it is time consuming. In practice, organizations may have insufficient time and staff to perform this extensive project. In the example described here, because there was insufficient time to hold a large number of interviews with patients to explore their perspectives on residence and care, only a patients' representative group was included in the study (step 1).

Furthermore, we did not double code our interview transcripts. However, the interview results were presented to the panel in the workshop, before work on scenarios started.

Organizations can take a practical approach to the method, and use as many elements as they have time for. However, they should be aware that a solid scenario analysis will eventually save time in developing policy plans of higher quality.

## Conclusions

The scenario study discussed here shows that, in connection with the future provision of residence types for people with mental health problems in Eindhoven, there should be a focus on investing in a socially supportive and responsible society and community-based care. This implies that more effort should be made to create social support systems and normalized living possibilities. It also means that cooperation between specialized mental health care providers, social care, public housing corporations, and local government is essential.

The scenario study has shown that working with scenarios provides policymakers of healthcare providers, public housing corporations, and local government with concrete guidelines for creating a shared vision of developments concerning a specific issue (in this study, the issue of types of residence for people with mental health problems), and with tools for understanding their implications for future strategy plans. Because the scenarios were constructed jointly by representatives from healthcare providers, public housing corporations, and local government, there is a strong possibility of regional support for strategy planning, of residence and care for mentally vulnerable people in the community by any of the organizations.

## Competing interests

The authors declare that they have no competing interests.

## Authors' contributions

All authors participated in the design of the study. JB carried out the document analysis and interviews. The workshop was organized and facilitated by JB and IB. The manuscript was initially composed by JB. IB and HvO contributed to the discussion and further improvement of the manuscript. All authors read and approved the final version of the manuscript.

## Pre-publication history

The pre-publication history for this paper can be accessed here:

http://www.biomedcentral.com/1472-6947/11/1/prepub
